# Oncocytoma-Related Gene Signature to Differentiate Chromophobe Renal Cancer and Oncocytoma Using Machine Learning

**DOI:** 10.3390/cells11020287

**Published:** 2022-01-15

**Authors:** Khaled Bin Satter, Paul Minh Huy Tran, Lynn Kim Hoang Tran, Zach Ramsey, Katheine Pinkerton, Shan Bai, Natasha M. Savage, Sravan Kavuri, Martha K. Terris, Jin-Xiong She, Sharad Purohit

**Affiliations:** 1 Center for Biotechnology and Genomic Medicine, Medical College of Georgia, Augusta University, 1120 15th Str., Augusta, GA 30912, USA; fbinsatter@augusta.edu (K.B.S.); ptran@augusta.edu (P.M.H.T.); lytran@augusta.edu (L.K.H.T.); kpinkerton@augusta.edu (K.P.); sbai@augusta.edu (S.B.); 2 Department of Pathology, Medical College of Georgia, Augusta University, 1120 15th Str., Augusta, GA 30912, USA; ZRAMSEY@augusta.edu (Z.R.); nsavage@augusta.edu (N.M.S.); skavuri@augusta.edu (S.K.); 3 Department of Urology, Medical College of Georgia, Augusta University, 1120 15th Str., Augusta, GA 30912, USA; mterris@augusta.edu; 4 Department of Obstetrics and Gynecology, Medical College of Georgia, Augusta University, 1120 15th Str., Augusta, GA 30912, USA; 5 Department of Undergraduate Health Professionals, College of Allied Health Sciences, Augusta University, 1120 15th Str., Augusta, GA 30912, USA

**Keywords:** chromophobe, oncocytoma, classification, machine learning, transcriptomic, gene signature

## Abstract

Publicly available gene expression datasets were analyzed to develop a chromophobe and oncocytoma related gene signature (COGS) to distinguish chRCC from RO. The datasets GSE11151, GSE19982, GSE2109, GSE8271 and GSE11024 were combined into a discovery dataset. The transcriptomic differences were identified with unsupervised learning in the discovery dataset (97.8% accuracy) with density based UMAP (DBU). The top 30 genes were identified by univariate gene expression analysis and ROC analysis, to create a gene signature called COGS. COGS, combined with DBU, was able to differentiate chRCC from RO in the discovery dataset with an accuracy of 97.8%. The classification accuracy of COGS was validated in an independent meta-dataset consisting of TCGA-KICH and GSE12090, where COGS could differentiate chRCC from RO with 100% accuracy. The differentially expressed genes were involved in carbohydrate metabolism, transcriptomic regulation by TP53, beta-catenin-dependent Wnt signaling, and cytokine (IL-4 and IL-13) signaling highly active in cancer cells. Using multiple datasets and machine learning, we constructed and validated COGS as a tool that can differentiate chRCC from RO and complement histology in routine clinical practice to distinguish these two tumors.

## 1. Introduction

Chromophobe renal cell carcinoma (chRCC) and oncocytoma (RO) are renal tumor types originating from alpha intercalated cells of the collecting ducts of the kidney [1,2] comprising 8–12% of all renal neoplasms [3,4,5,6]. Histologically, chRCC is composed of sheets of cells with well-defined cell borders that have darker cytoplasm than conventional clear cell carcinoma and peri-nuclear halos [6]. ChRCC, a malignant tumor, requires surgical intervention [3,5]. Histologically, RO has variable architecture and frequently consists of nests of tumor cells comprised of large, round, eosinophilic cells in loose connective tissue [6]. RO is a benign neoplasm and requires only periodic monitoring [6]. Gross morphology and histological similarities between the two tumors often pose difficulties in the classification of needle biopsy samples, which are the primary method of diagnosis of renal cancer [7]. Furthermore, medical imaging, such as CT-Scan or MRI, also fails to differentiate these tumors due to their similarity in appearance [8].

Immunohistochemical (IHC) markers for chRCC, such as cytokeratin 7, epithelial-mesenchymal antigen, and parvalbumin (PVALB) are commonly used in clinics by pathologists [9,10]. RO diagnosis is assisted by an IHC stain of cytokeratin 7, S100A1 [11], and kidney-specific cadherins [12]; however, the overlap between these markers in chRCC and RO makes it an ineffective method to distinguish these tumors [9,10,11]. Electron microscopy is the gold standard to differentiate the tumors, though the method is not feasible for routine clinical practice. Therefore, there is a need to identify additional markers to distinguish chRCC from RO.

Molecular diagnostic methods have been used to identify the specific genetic changes associated with disease and can be helpful for diagnostic and prognostic purposes [13]. Previous molecular studies on chRCC and RO proposed molecular markers such as parafibromin, aquaporin 6, and synaptogyrin 3 [14]. Additional molecular markers, such as AP1M2, MAL2, PROM2, PRSS8, FLJ20171 [15] and EGLN2 [16], are reported to be useful for distinguishing chRCC and RO in conjunction with the currently available IHC markers. The major drawbacks to these molecular diagnostic studies are the smaller sample sizes and the overlapping expression of genetic markers in chRCC and RO [14,15]. 

Here, we identified transcriptomic differences distinguishing chRCC from RO in a meta-dataset combined from multiple studies from the Gene Expression Omnibus (GEO) and ArrayExpress. We developed a 30-gene chromophobe and oncocytoma related gene signature (COGS), and elucidated pathway differences between chRCC and RO. We then implemented unsupervised machine learning (ML) algorithms and validated ML models to distinguish chRCC from RO.

## 2. Materials and Methods

### 2.1. Dataset Search and Selection

ChRCC and RO transcriptomic studies were identified in the Gene Expression Omnibus (GEO) and ArrayExpress (Table S1). Treatment-naïve samples and HG-U133plus 2 arrays were selected to create the discovery dataset. Based on the selection criteria, 6 studies (GSE11024, GSE11151, GSE12090, GSE2109, GSE8271, GSE19982) were chosen and their expression and phenotype data were downloaded with GEOquery [17]. The phenotype data were prepared from the downloaded files, and only chRCC, RO and normal renal tissue (N) arrays were selected in the data preparation steps. The data were preprocessed with probe selection, log transformation, and batch effect correction. 

### 2.2. Data Preprocessing and Probe-to-Gene Conversion

The best representative probe for each gene was identified in each study/array by implementing a probe selection algorithm [18] that scores individual probes based on the product of specificity, coverage, and robustness, and selects the highest scoring probe per gene. Finally, all the common probes across studies were subset into a data frame for further analysis (n = 15,875 probes).

The preprocessing steps included log transformation for GSE2109 and GSE11151 upon evaluation of the summary statistics. All the datasets were merged to create a data frame containing gene expression and phenotype information. Batch effects between the datasets were tested using principal component analysis (PCA) and were corrected using ComBat from “SVA” [19]. After batch correction, the data were re-evaluated for batch effects with PCA. This batch corrected dataset was used for all the subsequent analyses to generate COGS, differential expression, and validation, as described below.

### 2.3. Statistical Analysis

All statistical analyses in this study were performed using R language and environment for statistical computing [20], version 4.10. All *p*-values were two-sided and a *p* < 0.05 was considered significant.

#### 2.3.1. Unsupervised Learning Pipeline

We implemented an unsupervised machine learning algorithm with UMAP (Uniform Manifold Approximation and Projection) and Density-based UMAP to differentiate the tumors [21,22]. UMAP projects the high dimensional transcriptomic data into two-dimensional embedding while preserving local and global connectivity for each sample, resulting in similar sample groups together; the distance is inversely proportional to the similarity between the samples. Density-based UMAP (DBU) is the integration of UMAP, and density-based spatial cluster with the application of noise (DBSCAN) and is designed to run in iterations. The DBU took a random sample of genes, applied dimension reduction to project two-dimensional embedding, which was fed into DBACAN to identify the groups. The optimum parameters for UMAP are identified by running combinations of the hyperparameters (min_dist, n_neighbor, gene_count, metric). We ran a grid search for gene_count (25, 250, 1000, 5000, 10000), n_neighbor (5, 10, 20, 50), min_dist (0.01, 0.1, 0.2, 0.5, 0.99), and metric (Cosine, Manhattan, Euclidean) in all combinations to identify the best classification, by comparing inter- and intra-cluster distance in UMAP projection [23,24]. The chosen parameters are n_neighbors = 20, min_dist = 0.01, gene_count = 1000, and metric = “Manhattan”. DBU was iterated 1000 times with the selected parameters and the resulting two-dimensional UMAP coordinates were fed into a DBSCAN to identify the groups. The optimum parameter for DBSCAN was chosen using an elbow plot (eps = 2, Minimum points 5) from the “fpc” package [25]. Each iteration classified the sample set into groups, and these groups were compared across the iterations to develop the final classification. The final classification was computed with plurality voting, where a sample requires consensus from at least 70% of the iterations to be classified into that final group. If a sample fails to reach this threshold, it is considered “ambiguous”. The results of all the iterations were visualized as a consensus heatmap, generated using the package gplots [26]. An alluvial plot was generated with the ggalluvial package [27].

#### 2.3.2. Differential Expression and Network Analysis and Immune Cell Infiltration

Differential gene expression between chRCC, RO and normal kidney tissue was tested using LIMMA [28]. The output was fed into gene set enrichment analysis (GSEA) with fgsea [29], clusterprofiler [30] and ReactomePA [31] to identify networks and pathways involved in chRCC and RO [32]. Differential expression results were presented as a heatmap, generated using the package ComplexHeatmap [33].

#### 2.3.3. Chromophobe-Oncocytoma Gene Signature (COGS)

Thirty genes were selected to create a signature named the Chromophobe-Oncocytoma gene signature (COGS). The classification efficiency of COGS was evaluated using unsupervised learning (UMAP and DBU) and visualized as a heatmap. The sensitivity, specificity, accuracy, and AUC were calculated for each sample percentile. The genes with the highest sensitivity and specificity were initially selected [34]. This group was further categorized based on the expression levels at the various percentiles. Based on the literature reviewed, the genes found with higher fold change and relevance to chRCC or RO were given priority in the signature. 

#### 2.3.4. Validation Set Development and Signature Validation

The validation process for COGS consisted of creating a dataset by combining GSE12090 and TCGA-KICH (which was downloaded from the UCSC Xena browser and contains 65 chRCC samples). Next, batch effects were identified with PCA. Correction for batch related differences were conducted using the SVA package, and the removal of such effects was confirmed with PCA. UMAP and hierarchical clustering were implemented to evaluate COGS’s performance in differentiating chRCC and RO. The total TCGA renal cohort was also downloaded from UCSC Xena browser, to evaluate the signature’s ability to differentiate chRCC from other renal cancer types.

## 3. Results

### 3.1. Acquisition and Preprocessing of Datasets

**Figure 1:**
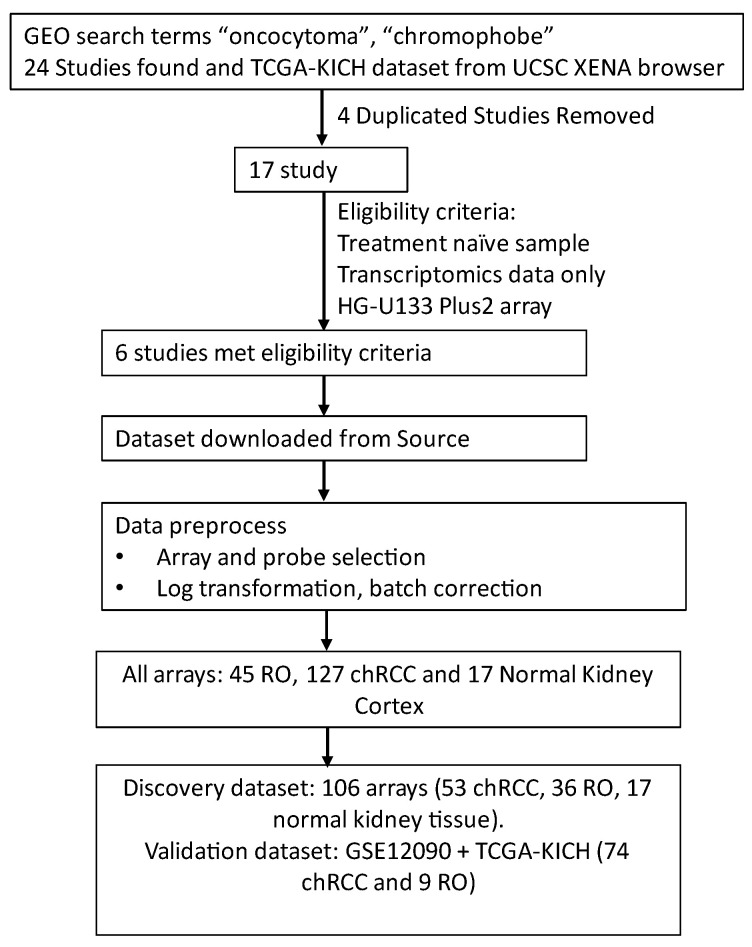
Flow chart depicting selection and preparation of chRCC and RO arrays from GEO for meta-analysis.

GEO and ArrayExpress were queried using the keywords “chromophobe and oncocytoma” on 6th June 2021; the query identified twenty-four records (Figure 1, Table 1). After removing duplicate entries, seventeen unique records were identified (Figure 1). We selected six studies based on following eligibility criteria: (1) patients were not treated at the time of sample collection, (2) gene expression data were available and (3) all gene expression studies were performed using Affymetrix HG-U133 plus2 array. Five studies (GSE11024, GSE2109, GSE19982, GSE8271, GSE11151) were combined to create a discovery dataset. The gene expression data for GSE12090 was retained for the validation study. Phenotype information was compiled from the downloaded datasets and all chRCC, RO and normal kidney tissue for further analysis. The final dataset consisted of 106 arrays, belonging to chRCC (n = 53), RO (n = 36) and normal kidney tissue (n = 17). The validation dataset GSE12090 contained chRCC (n = 9) and RO (n = 9) arrays, whereas TCGA-KICH solely contained chRCC (n = 65). The number of probes in each dataset differed among these arrays (Table 1). 

Since multiple probes on an array can represent a single gene, the best representative probe was identified for each gene from the HG-U133plus 2 array [18], identifying 15,875 probes in common for all datasets, with each probe representing a unique gene. 

**Figure 2:**
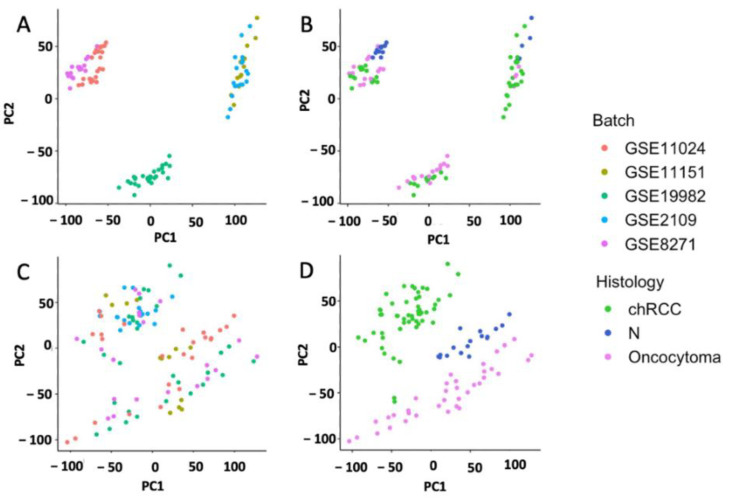
Quality control of the discovery dataset showing batch effects before and after correction. Principal component analysis showing differences in batch (**A**) is higher than difference in histology (**B**) for chromophobe (chRCC) and renal oncocytoma (RO) and normal kidney tissue arrays (N) before batch effect correction. After batch correction by empirical bayes (ComBat), histological differences (**D**) are higher than batch differences (**C**).

The discovery dataset was created by merging GSE11024, GSE11151, GSE9982, GSE2109 and GSE8271 (Table 1), in order to develop our unsupervised method and gene signature. After merging, the discovery dataset was evaluated for batch effects with PCA. We identified that batch-related differences were higher than histological differences (Figure 2A,B) between the studies. These batch-related effects were removed using ComBat from “SVA” and revisualized with PCA (Figure 2C,D).

### 3.2. Unsupervised Learning with UMAP and Density Based UMAP Largely Correlates with Histological Subtype

**Figure 3:**
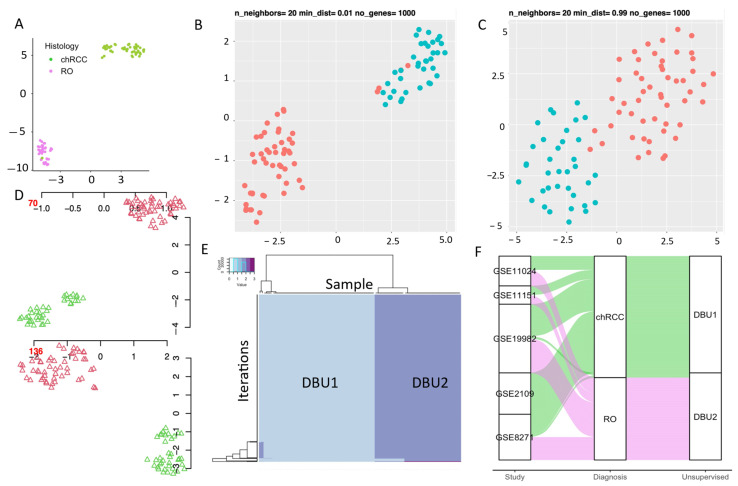
Implementation of unsupervised machine learning algorithm (UMLA) for differentiating chRCC and RO: (**A**) two dimension embedding for the whole genome (n = 15,875 genes) with UMAP, showing two clusters with high concordance with their histological classification; (**B**) representative map showing optimized final parameter for UMAP, best performing for maximum inter-cluster and minimum intra-cluster distance, red = chRCC, blue = RO; (**C**) representative map showing poorly fit parameters for UMAP analysis, red = “chRCC, blue = RO; (**D**) representative iterations for DBU (Iteration no 70, & 136). All 1000 iterations were tracked to determine final groups where support from > 70% iterations were needed, red triangles = cluster 1 in machine learning model, green triangles = cluster 2 in machine learning model (**E**) group consensus heatmap. Samples are presented in columns and iterations are in rows. Two colors (dark and light blue) represent two DBU groups based on the 1000 iterations of DBU with 1000 random genes in each iteration; (**F**) Sankey’s diagram tracking all samples from the study to DBU classification, color represents histology type (ChRCC = green, RO = pink). A total of 87/89 samples follow their histological classification with DBU.

We applied an unsupervised machine learning classification algorithm (UMLA) to evaluate its ability to differentiate chRCC and RO using the transcriptomics data. UMAP analysis using all genes (n = 15,875) projected two distinct clusters (Figure 3A). One cluster contains only chRCC samples (n = 51) and the 2nd cluster contains all RO (n = 36) and 2 chRCC samples, suggesting that these tumors are distinctive at transcriptomic level. To further evaluate the consistency and reproducibility of unsupervised learning, DBU was implemented for 1000 iterations. Optimum parameters for good vs. poor fit for a UMAP projections were n_neighbor = 20, gene_count = 1000, min_dist = 0.01, metric = “Manhattan” (Figure 3B,C). Each DBU iteration classified the samples into Groups (representative sample iterations, Figure 3D), and they are compared across the iterations for consistency. The final classification was computed with plurality voting which identified two groups (DBU1 and DBU2) (Figure 3E). The heatmap represent all the iterations (rows) and groups identified in each iteration (columns, color represents the group, Figure 3E). The DBU analysis was in 97.75% concordance with the histological classification (Figure 3F). Further analysis shows that DBU1 consisted only of chRCC samples (n = 51), whereas all RO and 2 chRCC were grouped in DBU2. These results show that unsupervised learning using UMAP and DBU is stable and can identify the differences between chRCC and RO in transcriptomic assays.

### 3.3. Development of COGS through Differential Expression, ROC, and Univariate Analysis

Our unsupervised machine learning was able to show distinct profiles for chRCC and RO by using 1000 randomly selected genes. To reduce the number of genes in the signature, we performed differential gene-expression analysis and receiver-operating characteristics analysis to identify candidate genes for differentiating between these two tumors, which can be used for development of chRCC and RO related gene signature (COGS). 

Differential expression analysis identified 8411 out of 15,875 genes as differentially expressed between chRCC and RO (Figure S1). Out of the 8411 genes, 299 genes have at least two-fold differences between the two tumor types. AUC analysis of these 299 genes identified 194 genes with an AUC of 0.9 or higher between the tumors. Hierarchical clustering with these top 194 genes showed a clear distinction between chRCC and RO, similar to unsupervised learning, and these candidate genes proceeded to the next phase for signature development (Table S2). 

**Figure 4:**
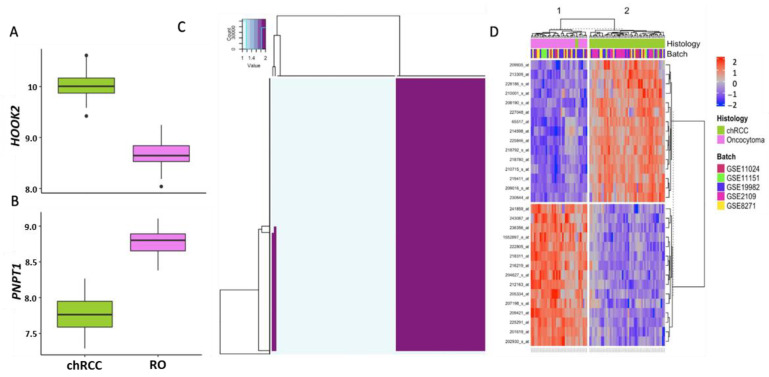
Gene selection and unsupervised model consistency: (**A**,**B**) boxplot showing log2-transformed expression values for (y-axis) for two representative genes (*HOOK2* and *PNPT1*) from COGS in chRCC (green) and RO (magenta), outliers are represented with points (black); (**C**) group consensus heatmap by DBU with 20 random genes from GS30, showing a consistent classification with unsupervised models; (**D**) heatmap of COGS in meta-analysis showing expression differences between the subtypes.

Genes with maximum diagnostic utility were identified based on sensitivity, specificity, AUROC, and accuracy at all percentiles for the candidate genes. We identified that 84 of the 194 genes have a sensitivity > 0.91, specificity > 0.91, AUROC > 0.92, and accuracy > 0.92. In prioritizing the genes with expression overlap between the tumors, a greater fold change, significance at more inter-percentile fold change, and relevance to cancers, thirty genes were finally selected as a gene signature, called “COGS”. (Figure 4A,B and Figure S2, Table 2).

COGS was assessed for accuracy, stability and robustness using 1000 iterations of the DBU models on the discovery dataset (accuracy 97.8%) (Figure 4C). Models with fewer than 30 genes had decreased accuracy; hence COGS consists of 30 genes (Figure S3). DBU with COGS has an accuracy of 97.8% in plurality voting for 1000 iterations; this is comparable to the 1000 gene model and confirms its ability to recapitulate the difference between the tumors to a similar degree to random 1000-gene models (Figure 3C) and whole-genome models (Figure 3B). In total, 51/53 of the chRCC and 36/36 of the RO samples are correctly classified with these models. Two chRCC samples are classified as RO, similar to the 1000-gene models.

### 3.4. Pathway Analysis Identified Enriched Carbohydrate Metabolism in chRCC and Deviation of Warburg Effect in Both Tumors

Functional differences between chRCC and RO were analyzed using differentially expressed genes in gene set enrichment analysis. We identified 67 pathways between the tumors (adjusted *p* value < 0.05, Benjamini-Hochberg correction) out of 218 pathways (Table S3). Notably, enriched pathways were carbohydrate metabolism, transcriptomic regulation by *TP53*, *beta-catenin* independent WNT signaling, diseases of signal transduction by growth factor receptors and second messengers, interleukin 4 and 13 signaling, glycosaminoglycan metabolism, and extracellular matrix organization (Figure S4).

**Figure 5:**
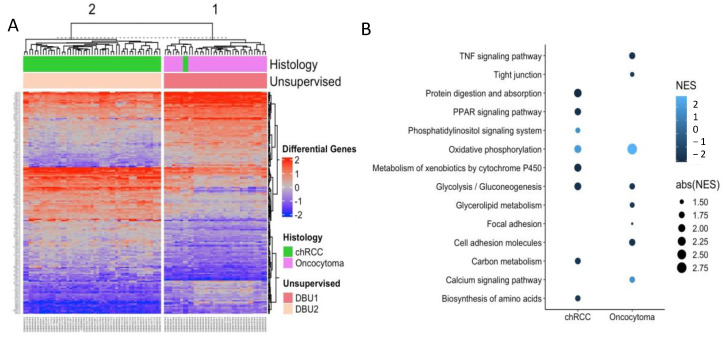
Molecular and pathway analysis: (**A**) top 194 Differentially expressed genes (candidate genes) between chRCC and RO. Genes are presented in rows and arrays are in columns; (**B**) bubble plot showing top upregulated pathways in chRCC and RO (x-axes) when compared to normal kidney tissue from gene set enrichment analysis for canonical pathways for differentially expressed genes. Y-axes represent different canonical pathways and size of the bubble represents normalized enrichment score.

KEGG pathway analysis identified 19 pathways enriched in chRCC from the normal kidney cortex. Upregulated pathways include oxidative phosphorylation (NES 1.92, adjusted *p*-value 1.82 × 10^−4^) and phosphatidylinositol signaling (NES 1.59, BH adjusted *p* value 3.38 × 10^−3^) (Figure 5B and Table S4). KEGG pathway analysis on RO, as compared to normal the kidney cortex, identified 41 pathways significantly enriched (BH adjusted, *p*-value < 0.05), with upregulation of the oxidative phosphorylation and calcium signaling pathways (NES 2.8 and 1.6, *p*-value 3.74 × 10^−17^). Downregulated pathways in RO include mineral absorption (NES = −2.2), cell adhesion molecules (NES = −1.8), glycolysis and gluconeogenesis (NES = −1.71), and *TNF* signaling pathway (NES = −1.79) (Figure 5B and Table S5). 

### 3.5. Validation of COGS in a Microarray and RNA-Seq Combined Meta-Dataset

**Figure 6:**
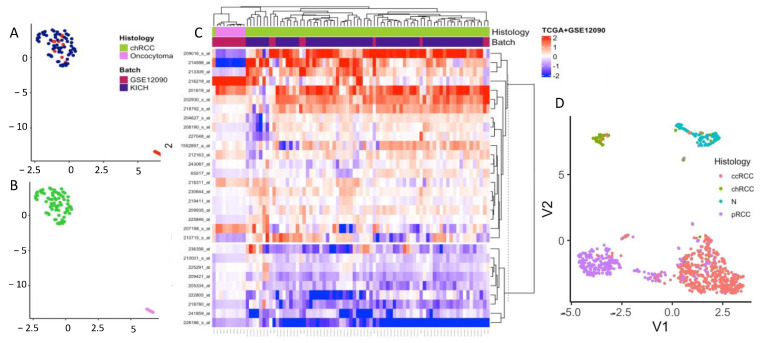
COGS validation on RNA-Seq (TCGA-KICH) and micro-array dataset (GSE12090): (**A**) two-dimensional embedding plot with UMAP using COGS showing no batch effect between the studies in the validation dataset; (**B**) sample plot with histology annotation shows distinct clusters for chRCC (n = 74) and RO (n = 9) with COGS; (**C**) unsupervised hierarchical clustering with COGS for validation dataset (**D**) UMAP of TCGA renal cohort showing distinct cluster for chRCC samples (lime green) for COGS.

We validated the performance of COGS to distinguish chRCC from RO using a validation dataset consisting of a microarray (GSE12090, 9 chRCC and 9 RO) and RNA-Seq data (TGCA-KICH, 65 chRCC). Batch differences were visualized using PCA (Figure S5A,B) and were corrected by ComBat from the SVA package (Figure S5C,D). UMAP and heatmap analysis of the validation meta-dataset showed two clusters without notable batch effects (Figure 6A). ChRCC and RO both formed their own clusters with UMAP and hierarchical cluster analysis (Figure 6B,C). This showed COGS’s ability to accurately differentiate chRCC and RO from each other and to a comparable degree as 1000-gene models (Figure 3E,F) and whole genome models (Figure 3A) in the validation dataset. This also demonstrates the applicability of the gene signature to microarray as well as RNA-Seq. 

COGS was able to identify chRCC samples in a TCGA-pan-renal cohort. We implemented UMAP in the pan-renal COGS expression to check COG’s ability to identify chRCC samples from other renal cancer types. UMAP representation shows a chRCC cluster, and 62/65 chRCC samples are grouped together (Figure 6D). This result shows that COGS can identify chRCC samples, even amongst other renal cancer types.

## 4. Discussion

In this study, we developed a machine learning approach to differentiate chRCC from RO using transcriptomic data. We showed here that the two kidney cancers, chRCC and RO have a distinct transcriptomic. We built an unsupervised machine learning pipeline to differentiate the tumors using these. We developed COGS, a gene signature with thirty genes, with highest diagnostic utility (Figure 3A). Unsupervised classification with COGS provided consistent classification (accuracy 97.8% in the discovery dataset, and 100% in the validation dataset) (Figure 4C and Figure 5B). These results show potential for clinical use for differentiating chRCC from RO, and can be employed with microarray or RNA-Seq-based assays. In addition, these assays do not require a large amount of tissue, making this a suitable method for classifying a needle biopsy sample. Current diagnostic approaches using histology with or without immunohistochemical staining are often insufficient to confirm diagnosis. We show that COGS can bridge the gap between the two pathologies through an ML-based approach, and that COGS can complement current clinical workflow by confirming the diagnosis in histologically uncertain cases.

IHC and special stain markers for distinguishing chRCC and RO—including CK7, S100A1, CD117 (CKIT), kidney specific cadherins, KAI, cyclin D1, and Hale’s colloidal iron—are currently used to differentiate the tumors. Current IHC markers lack the sensitivity and specificity needed to make a confirmed diagnosis. Immune-histochemical analyses of CK7 and S100A1 have a broad range of sensitivity, and specificity of 80–100% and 70–92% (for chRCC), and 80–100% and 70–92% (for RO), which could be improved [10,35]. Hale colloidal iron is also insufficient in biopsy samples because of focal positivity seen in RO [36]. In comparison, our unsupervised ensemble models have a sensitivity and specificity of 97.8% and 94.7% for chRCC. This is markedly better than current IHC markers used for distinguishing chRCC from RO. Five genes (AP1M2, AQP6, HOOK2, CLDN, ESRP1) that were identified in our panel, have previously been reported as candidate markers in the literature, which is further validation of their importance in these tumors [15,37].

Signal transduction by growth factor receptor, TP53 transcriptional regulation, platelet activation, signaling, and aggregation are among the top differing functional pathways between chRCC and RO on Reactome pathway analysis. KEGG pathway analysis on chRCC identified that calcium signaling, carbon and glycolipid metabolism, PPAR signaling pathway, and TNF signaling pathways (Figure 4B) vary in expression from normal kidney tissue. ChRCC and RO both showed upregulated oxidative phosphorylation with decreased glycolysis and gluconeogenesis suggesting deviation from the Warburg effect, which is commonly present in renal tumors.

This study has few limitations. First it is based on the gene expression differences profiled on bulk tumors containing heterogenous cells, viz. tumor, stromal and infiltrated immune cells. Therefore, additional studies may be required to address spatiotemporal and functional differences at a cellular level. Another minor limitation of the study lies in the selection bias inherent to retrospective analyses. Multiple studies were included in the discovery meta-dataset to reduce the potential selection bias in the construction of COGS. Retrospective validation was also performed to confirm the efficacy of COGS in the classification of ChRCC and RO, hence another instance of selection bias may be present in the validation phase of this study. To address this limitation, wet lab experiments using clinical specimens will be designed to prospectively validate COGS at our institution. 

The strengths of our study are a relatively large gene expression meta-dataset for chRCC- RO combined with validation of COGS. Another strength of our study is the application of the ensemble unsupervised machine learning model, which showed consistent classification for chRCC and RO. This algorithm out-performed the current IHC-based methods with 97.8% accuracy in the discovery meta-dataset [10]. Validation with UMAP and hierarchical clustering on GSE12090-KICH(TCGA) have an accuracy of 100%. Our results showed that machine learning models in transcriptomics out-perform current methods and can complement the current diagnostic pipeline in difficult cases. 

## 5. Conclusions

We identified transcriptomic differences distinguishing chRCC from RO in a meta-dataset combined from multiple studies. From the gene expression differences, we implemented machine learning (ML) algorithms and developed and validated COGS, a 30-gene transcriptomic signature, and ML models to distinguish chRCC from RO.

## Figures and Tables

**Table 1:** GEO datasets selected for chromophobe renal cell carcinoma (chRCC) and renal oncocytoma (RO) classification as discovery and validation datasets. Table contains details on number of probes, total number of arrays in the study and number of arrays selected under chRCC, RO and normal kidney tissue.

GEO Accession ID	Number of Probes	Total Number of Arrays/Study	Number of Arrays Selected		
			RO	ChRCC	Normal Kidney
GSE11024	17,700	79	7	6	12
GSE11151	54,676	67	4	4	5
GSE19982	54,676	30	15	15	0
GSE8271	54,676	34	10	10	0
GSE2109	17,232	2158	0	18	0
TCGA-KICH	60,483	89	0	65	24
GSE12090	54,676	18	9	9	0

**Table 2:** List of thirty genes combined to create COGS signature for distinguishing chRCC from RO. Data presented is for discovery dataset to show sensitivity-specificity, accuracy, area under the receiver operator curve (AUROC) and log fold change (FC) for discovery meta-dataset.

Gene	Optimum Cutpoint	Accuracy	Sensitivity	Specificity	AUROC	FC *	Adj *p*-Val
*AP1M2*	8.87	0.98	0.96	1.00	1.00	4.48	4.63 × 10^−26^
*AQP6*	8.29	0.98	0.94	1.00	1.00	36.98	2.61 × 10^−32^
*ATP2C1*	8.02	0.94	0.91	0.97	0.99	2.76	1.07 × 10^−21^
*BSPRY*	7.82	1.00	1.00	1.00	1.00	3.52	1.73 × 10^−26^
*CLDN8*	10.50	0.93	0.91	0.94	0.97	42.73	1.12 × 10^−19^
*DNAI3*	5.23	0.94	0.94	0.94	0.98	3.32	9.59 × 10^−20^
*ELMO3*	7.86	1.00	1.00	1.00	1.00	3.07	1.85 × 10^−28^
*ESRP1*	7.99	0.99	0.98	1.00	1.00	10.32	5.01 × 10^31^
*HOOK2*	9.42	1.00	1.00	1.00	1.00	3.84	5.12 × 10^−36^
*ITGB3*	6.79	0.99	1.00	0.98	1.00	3.68	3.84 × 10^−23^
*KCNG3*	5.49	1.00	1.00	1.00	1.00	2.80	9.52 × 10^−24^
*KIDINS220*	9.19	1.00	1.00	1.00	1.00	2.93	3.08 × 10^−28^
*KRT7*	7.72	0.96	0.94	1.00	0.98	55.34	4.83 × 10^−27^
*LAMA1*	5.87	0.94	0.96	0.92	0.97	6.88	1.60 × 10^−21^
*LIMS1*	10.35	0.94	0.94	0.94	0.97	3.33	3.70 × 10^−16^
*LRFN5*	6.31	0.96	0.94	1.00	0.99	8.02	3.03 × 10^−23^
*LSR*	8.72	1.00	1.00	1.00	1.00	3.46	1.51 × 10^−34^
*MANEA*	5.70	0.99	1.00	0.98	1.00	3.04	1.26 × 10^−28^
*MAP4K3*	8.91	1.00	1.00	1.00	1.00	5.22	2.91 × 10^−31^
*MSH2*	6.54	0.99	1.00	0.98	1.00	3.63	2.68 × 10^−29^
*NDUFS1*	9.18	0.99	0.97	1.00	0.99	3.16	2.79 × 10^−27^
*PLCL1*	8.52	0.96	0.94	0.98	0.95	6.05	6.35 × 10^−19^
*PLCL2*	7.79	0.98	0.96	1.00	1.00	5.78	2.48 × 10^−27^
*PNPT1*	8.38	1.00	1.00	1.00	1.00	2.80	5.05 × 10^−34^
*PRDX3*	11.59	0.98	0.97	0.98	1.00	4.02	4.40 × 10^−26^
*RSPO3*	6.89	0.98	0.96	1.00	0.99	6.29	4.17 × 10^−23^
*S100A1*	8.43	0.95	1.00	0.91	0.98	3.70	3.90 × 10^−19^
*SOCS1*	7.32	0.92	0.91	0.92	0.97	3.59	1.05 × 10^−18^
*SPINT2*	10.59	1.00	1.00	1.00	1.00	3.53	4.32 × 10^−30^
*SUCLA2*	9.71	0.99	1.00	0.98	1.00	3.68	1.09 × 10^−26^

* FC: fold change, AUC: area under the curve.

## Data Availability

Data for this study are publicly available and can be downloaded from the gene expression omnibus (GEO) or ArrayExpress website using the accession number. Histopathology and other clinical information are also available with the datasets.

## Supplementary Materials

The following supporting information can be downloaded at: https://www.mdpi.com/article/10.3390/cells11020287/s1, Figure S1: Differential expression analysis between chRCC and RO tumor types using 15,875 genes. Figure S2: Dot plots for each gene in COGS for classification in chRCC (green dots) and renal oncocytoma (magenta dots). Figure S3: Model performance from whole genome to 10-gene model. Accuracy was checked with density based UMAP classification. Figure S4: Reactome pathways analysis between chRCC and RO for all genes (15,875 genes) showing enrichment of specific pathways. Figure S5: Principal component analysis showing batch effects (A,B) and after correction (C,D) on the validation dataset. Table S1: Micro-array and RNA-Seq data sets used in the study and the web links to access them. Table S2: List of top 194 differentially expressed genes between chRCC and RO. Table S3: Reactome Pathway analysis between chRCC and RO using all genes (n = 15,875). Table S4: KEGG pathway analysis of genes (n = 15,875) between chRCC and normal kidney. Table S5: KEGG pathway analysis of genes (n = 15,875) between RO and Normal Kidney.
