# Supplementary material for: Oncocytoma-Related Gene Signature to Differentiate Chromophobe Renal Cancer and Oncocytoma Using Machine Learning
Source: Cells. 2022 Jan 15;11(2):287. doi: 10.3390/cells11020287 (PMC8774230; doi:10.3390/cells11020287)
Supplement: Supplementary file 1 [file cells-11-00287-s001.zip › cells-1551407-Supplementary.pdf]

Table S1: Micro-array and RNA-Seq data sets used in the study and the web links to access them.

Study	Access Links	Date Accessed
GSE11024	https://www.ncbi.nlm.nih.gov/geo/query/acc.cgi?acc=GSE11024	6/6/2021
GSE11151	https://www.ncbi.nlm.nih.gov/geo/query/acc.cgi?acc=GSE11151	6/6/2021
GSE19982	https://www.ncbi.nlm.nih.gov/geo/query/acc.cgi?acc=GSE19982	6/6/2021
GSE8271	https://www.ncbi.nlm.nih.gov/geo/query/acc.cgi?acc=GSE8271	6/6/2021
GSE2109	https://www.ncbi.nlm.nih.gov/geo/query/acc.cgi?acc=GSE2109	6/6/2021
GSE12090	https://www.ncbi.nlm.nih.gov/geo/query/acc.cgi?acc=GSE12090	6/6/2021
TCGA-KICH	https://xenabrowser.net/datapages/?dataset=TCGA-KICH.htseq_fpkm-uq.tsv&host=https%3A%2F%2Fgdc.xenahubs.net&removeHub=https%3A%2F%2Fxena.treehouse.gi.ucsc.edu%3A443	6/6/2021

Table S2: List of top 194 differentially expressed genes between chRCC and RO.

Probe ID	SYMBOL	ENTREZ	logFC	AveExpr	t	P.Value	adj.P.Val	B	AUC.x	optimal_cutp	sum_sens_sp	acc	sensitivity	specificity
1552797_s_a	PROM2	150696	-1.35	7.67	-11.83	7.63E-20	2.74E-18	34.62	0.98	7.76	1.83	0.90	0.83	1.00
1552897_a_a	KCNG3	170850	1.01	5.41	15.11	4.14E-26	5.31E-24	48.96	1.00	5.49	2.00	1.00	1.00	1.00
1557137_at	TMEM17	200728	1.30	6.18	15.60	5.21E-27	8.09E-25	51.02	0.99	6.07	1.94	0.98	0.94	1.00
1557146_a_a	SSTR5-AS1	146336	1.25	7.62	10.50	3.71E-17	8.53E-16	28.47	0.96	7.42	1.78	0.89	0.89	0.89
200662_s_at	TOMM20	9804	1.00	10.20	11.93	4.88E-20	1.82E-18	35.06	0.95	10.26	1.85	0.93	0.89	0.96
200978_at	MDH1	4190	1.07	11.82	14.83	1.35E-25	1.50E-23	47.79	1.00	11.93	1.98	0.99	1.00	0.98
201141_at	GPNMB	10457	-3.04	10.16	-12.05	2.77E-20	1.08E-18	35.63	0.95	9.61	1.83	0.92	0.94	0.89
201212_at	LGMN	5641	-1.15	11.21	-9.55	3.15E-15	5.18E-14	24.05	0.91	10.95	1.73	0.87	0.89	0.83
201502_s_at	NFKBIA	4792	-1.45	10.75	-10.05	3.04E-16	6.01E-15	26.37	0.93	10.83	1.73	0.86	0.79	0.94
201619_at	PRDX3	10935	1.43	11.28	16.70	5.94E-29	1.50E-26	55.46	1.00	11.59	1.95	0.98	0.97	0.98
201721_s_at	LAPTM5	7805	-1.02	9.21	-10.41	5.57E-17	1.24E-15	28.06	0.94	8.85	1.81	0.92	1.00	0.81
201843_s_at	EFEMP1	2202	2.70	8.93	15.77	2.59E-27	4.37E-25	51.72	0.98	9.59	1.87	0.94	0.89	0.98
201849_at	BNIP3	664	1.06	9.90	13.11	2.36E-22	1.38E-20	40.36	0.98	9.83	1.91	0.95	1.00	0.91
202432_at	PPP3CB	5532	1.21	9.32	15.20	2.77E-26	3.71E-24	49.36	0.98	9.39	1.90	0.95	0.92	0.98
202502_at	ACADM	34	1.78	10.43	14.14	2.55E-24	2.29E-22	44.87	0.97	10.55	1.90	0.95	0.94	0.96
202525_at	PRSS8	5652	-2.75	9.52	-16.21	4.33E-28	8.82E-26	53.49	0.99	9.66	1.90	0.95	0.96	0.94
202749_at	GET1	7485	1.26	8.85	10.00	3.87E-16	7.46E-15	26.14	0.92	9.03	1.76	0.89	0.78	0.98
202826_at	SPINT1	6692	-2.03	9.86	-16.45	1.64E-28	3.89E-26	54.46	0.99	9.98	1.95	0.98	0.98	0.97
202930_s_at	SUCLA2	8803	1.35	9.63	17.13	1.10E-29	3.30E-27	57.13	1.00	9.71	1.98	0.99	1.00	0.98
202962_at	KIF13B	23303	-1.30	10.31	-16.89	2.82E-29	7.73E-27	56.20	1.00	10.32	1.95	0.98	0.98	0.97
203138_at	HAT1	8520	1.14	8.21	9.58	2.80E-15	4.65E-14	24.17	0.94	8.29	1.75	0.88	0.86	0.89
203196_at	ABCC4	10257	1.54	7.49	15.72	3.18E-27	5.27E-25	51.51	0.99	7.93	1.94	0.98	0.94	1.00
203303_at	DYNLT3	6990	1.49	10.98	12.44	4.70E-21	2.11E-19	37.39	0.97	11.29	1.91	0.95	0.97	0.94
203474_at	IQGAP2	10788	-1.02	8.91	-8.86	8.36E-14	1.11E-12	20.80	0.91	8.79	1.77	0.89	0.94	0.83
203476_at	TPBG	7162	-1.49	9.11	-10.87	6.51E-18	1.69E-16	30.20	0.94	9.31	1.77	0.88	0.85	0.92
203608_at	ALDH5A1	7915	1.21	7.73	11.52	3.11E-19	1.01E-17	33.22	0.96	7.71	1.85	0.92	1.00	0.85
203711_s_at	HIBCH	26275	1.14	9.35	8.37	8.33E-13	9.34E-12	18.52	0.90	9.59	1.70	0.86	0.81	0.89
203717_at	DPP4	1803	1.78	8.50	9.89	6.42E-16	1.19E-14	25.63	0.91	8.83	1.86	0.94	0.86	1.00
203860_at	PCCA	5095	1.11	10.92	9.57	2.90E-15	4.79E-14	24.13	0.94	10.80	1.82	0.90	0.97	0.85
203881_s_at	DMD	1756	1.21	7.29	10.06	2.93E-16	5.81E-15	26.41	0.93	7.33	1.75	0.88	0.86	0.89
203892_at	WFDC2	10406	-1.34	6.96	-12.29	9.48E-21	3.94E-19	36.69	0.97	6.99	1.82	0.90	0.87	0.94
204037_at	LPAR1	1902	1.07	8.19	12.46	4.39E-21	1.97E-19	37.46	0.97	8.17	1.87	0.94	0.92	0.96
204122_at	TYROBP	7305	-1.02	8.46	-10.09	2.51E-16	5.01E-15	26.56	0.95	8.27	1.78	0.89	0.91	0.86
204254_s_at	VDR	7421	-1.24	8.45	-15.39	1.29E-26	1.87E-24	50.12	0.99	8.35	1.96	0.98	0.96	1.00
204273_at	EDNRB	1910	1.24	7.99	9.82	8.81E-16	1.60E-14	25.32	0.94	8.16	1.74	0.87	0.89	0.85
204286_s_at	PMAIP1	5366	-1.43	6.06	-8.91	6.71E-14	9.03E-13	21.01	0.93	5.73	1.83	0.92	0.94	0.89
204485_s_at	TOM1L1	10040	1.36	9.09	13.06	3.00E-22	1.73E-20	40.13	0.98	9.42	1.90	0.95	0.94	0.96
204627_s_at	ITGB3	3690	1.29	6.91	15.20	2.81E-26	3.71E-24	49.35	1.00	6.79	1.98	0.99	1.00	0.98
204653_at	TFAP2A	7020	-1.22	6.65	-9.65	1.97E-15	3.35E-14	24.52	0.90	6.47	1.69	0.86	0.94	0.75
204675_at	SRD5A1	6715	-1.04	6.96	-12.73	1.28E-21	6.61E-20	38.69	0.98	6.83	1.91	0.95	0.94	0.97
204719_at	ABCA8	10351	2.94	7.05	13.31	9.59E-23	6.09E-21	41.26	0.96	7.29	1.86	0.93	0.94	0.91
204821_at	BTN3A3	10384	1.01	6.51	10.41	5.59E-17	1.24E-15	28.06	0.96	6.56	1.84	0.93	0.86	0.98
204849_at	TCFL5	10732	1.28	7.60	10.10	2.37E-16	4.75E-15	26.62	0.94	7.75	1.80	0.90	0.89	0.91
205075_at	SERPINF2	5345	1.40	8.60	9.75	1.26E-15	2.21E-14	24.96	0.91	8.90	1.70	0.87	0.72	0.98
205139_s_at	UST	10090	1.26	7.23	8.65	2.26E-13	2.78E-12	19.81	0.90	7.42	1.71	0.86	0.83	0.87
205173_x_at	CD58	965	1.23	7.62	10.13	2.08E-16	4.24E-15	26.75	0.94	7.65	1.75	0.88	0.86	0.89

205200_at	CLEC3B	7123	1.95	9.97	9.37	7.48E-15	1.16E-13	23.19	0.90	11.21	1.67	0.86	0.67	1.00
205202_at	PCMT1	5110	1.04	9.23	16.28	3.31E-28	6.92E-26	53.76	0.99	9.33	1.95	0.98	0.97	0.98
205302_at	IGFBP1	3484	3.42	5.55	11.21	1.36E-18	4.02E-17	31.76	0.94	4.60	1.77	0.87	1.00	0.77
205334_at	S100A1	6271	1.25	8.53	11.72	1.24E-19	4.32E-18	34.13	0.97	8.43	1.91	0.95	1.00	0.91
205352_at	SERPINI1	5274	-1.27	8.80	-8.70	1.80E-13	2.25E-12	20.04	0.90	8.64	1.85	0.93	0.96	0.89
205549_at	PCP4	5121	-2.40	9.88	-8.99	4.56E-14	6.26E-13	21.40	0.91	8.93	1.74	0.88	0.96	0.78
205573_s_at	SNX7	51375	1.39	8.59	12.83	8.28E-22	4.44E-20	39.12	0.97	8.54	1.82	0.90	0.94	0.87
205590_at	RASGRP1	10125	-1.66	7.14	-10.10	2.36E-16	4.74E-15	26.63	0.94	7.03	1.79	0.89	0.87	0.92
205609_at	ANGPT1	284	-1.37	8.31	-9.76	1.21E-15	2.13E-14	25.01	0.94	8.51	1.80	0.89	0.85	0.94
205632_s_at	PIP5K1B	8395	-1.12	7.68	-10.32	8.49E-17	1.83E-15	27.64	0.91	7.52	1.81	0.92	1.00	0.81
205799_s_at	SLC3A1	6519	2.26	11.28	9.19	1.77E-14	2.58E-13	22.34	0.94	11.59	1.90	0.95	0.94	0.96
205857_at	SLC18A2	6571	1.77	5.94	11.08	2.41E-18	6.82E-17	31.18	0.95	5.86	1.84	0.92	0.94	0.89
206307_s_at	FOXD1	2297	2.13	7.05	9.12	2.45E-14	3.48E-13	22.01	0.90	7.05	1.75	0.87	0.94	0.81
206529_x_at	SLC26A4	5172	-1.51	6.07	-7.10	3.07E-10	2.31E-09	12.67	0.90	5.81	1.73	0.86	0.79	0.94
206632_s_at	APOBEC3B	9582	-1.01	5.80	-8.69	1.86E-13	2.32E-12	20.00	0.91	5.53	1.68	0.84	0.87	0.81
206683_at	ZNF165	7718	-1.78	6.75	-11.81	8.38E-20	2.98E-18	34.52	0.94	6.26	1.84	0.93	0.98	0.86
207177_at	PTGFR	5737	2.38	5.67	11.01	3.34E-18	9.22E-17	30.86	0.94	5.75	1.74	0.88	0.78	0.96
207198_s_at	LIMS1	3987	1.17	10.17	10.58	2.48E-17	5.84E-16	28.87	0.96	10.35	1.88	0.94	0.94	0.94
207655_s_at	BLNK	29760	1.08	10.07	9.64	2.07E-15	3.49E-14	24.47	0.93	10.20	1.73	0.87	0.86	0.87
208190_s_at	LSR	51599	-1.21	8.77	-22.33	6.74E-38	2.14E-34	75.83	1.00	8.72	2.00	1.00	1.00	1.00
208383_s_at	PCK1	5105	3.74	10.14	9.42	5.99E-15	9.41E-14	23.41	0.90	12.01	1.70	0.87	0.72	0.98
208671_at	SERINC1	57515	1.33	10.62	14.39	8.65E-25	8.58E-23	45.94	0.99	10.75	1.93	0.96	0.97	0.96
209016_s_at	KRT7	3855	-4.00	8.56	-16.97	2.09E-29	5.94E-27	56.50	0.99	7.72	1.94	0.96	0.94	1.00
209183_s_at	DEPP1	11067	1.02	8.54	9.66	1.93E-15	3.28E-14	24.54	0.92	8.89	1.70	0.87	0.72	0.98
209406_at	BAG2	9532	1.08	5.47	12.03	3.02E-20	1.16E-18	35.54	0.98	5.54	1.87	0.94	0.92	0.96
209421_at	MSH2	4436	1.28	6.44	19.51	1.31E-33	1.16E-30	66.08	1.00	6.54	1.98	0.99	1.00	0.98
209465_x_at	PTN	5764	-1.78	8.65	-9.44	5.31E-15	8.40E-14	23.53	0.95	8.15	1.85	0.93	0.94	0.92
209602_s_at	GATA3	2625	-2.04	6.92	-9.50	4.08E-15	6.57E-14	23.79	0.93	7.05	1.73	0.86	0.79	0.94
209619_at	CD74	972	-1.53	11.31	-11.30	8.62E-19	2.62E-17	32.21	0.94	10.93	1.78	0.90	0.98	0.81
209956_s_at	CAMK2B	816	-1.30	6.47	-9.55	3.26E-15	5.35E-14	24.02	0.95	6.44	1.87	0.93	0.89	0.97
210001_s_at	SOCS1	8651	-1.24	7.47	-11.93	4.79E-20	1.79E-18	35.08	0.96	7.32	1.83	0.92	0.91	0.92
210108_at	CACNA1D	776	-1.00	7.25	-9.78	1.08E-15	1.93E-14	25.11	0.94	6.85	1.76	0.89	0.96	0.81
210715_s_at	SPINT2	10653	-1.23	10.56	-18.80	1.78E-32	1.09E-29	63.50	1.00	10.59	2.00	1.00	1.00	1.00
211990_at	HLA-DPA1	3113	-1.46	10.71	-9.18	1.85E-14	2.68E-13	22.29	0.92	10.56	1.79	0.89	0.87	0.92
212070_at	ADGRG1	9289	-1.17	11.06	-12.31	8.66E-21	3.62E-19	36.78	0.96	11.22	1.84	0.92	0.87	0.97
212163_at	KIDINS220	57498	1.06	8.99	18.44	7.01E-32	3.97E-29	62.15	1.00	9.19	2.00	1.00	1.00	1.00
212453_at	KIFBP	26128	1.00	8.63	15.50	8.13E-27	1.19E-24	50.58	1.00	8.81	1.96	0.98	1.00	0.96
212503_s_at	DIP2C	22982	1.20	8.78	14.33	1.12E-24	1.08E-22	45.69	0.98	8.66	1.88	0.94	0.94	0.94
212818_s_at	ASB1	51665	1.70	6.69	11.31	8.31E-19	2.54E-17	32.24	0.94	6.79	1.81	0.92	0.83	0.98
212950_at	ADGRF5	221395	1.67	11.56	7.80	1.22E-11	1.14E-10	15.86	0.90	12.38	1.76	0.89	0.81	0.96
213085_s_at	WWC1	23286	-1.54	8.95	-9.95	4.80E-16	9.13E-15	25.92	0.91	8.88	1.77	0.89	0.94	0.83
213172_at	TTC9	23508	1.58	6.45	8.99	4.56E-14	6.26E-13	21.40	0.91	6.24	1.63	0.81	0.89	0.74
213285_at	TMEM30B	161291	-3.50	8.66	-14.96	7.60E-26	9.14E-24	48.36	0.97	8.98	1.82	0.92	0.96	0.86
213309_at	PLCL2	23228	-1.68	7.83	-16.11	6.57E-28	1.32E-25	53.08	1.00	7.79	1.96	0.98	0.96	1.00
213624_at	SMPDL3A	10924	1.30	8.96	10.17	1.69E-16	3.47E-15	26.96	0.94	9.17	1.83	0.92	0.92	0.91
213929_at	EXPH5	23086	-1.20	9.51	-9.27	1.20E-14	1.80E-13	22.72	0.91	9.28	1.69	0.86	0.94	0.75
214043_at	PTPRD	5789	-1.12	7.68	-7.63	2.72E-11	2.40E-10	15.07	0.90	7.19	1.71	0.87	0.94	0.78
214079_at	DHRS2	10202	-3.30	7.54	-8.76	1.37E-13	1.75E-12	20.31	0.95	6.55	1.84	0.92	0.89	0.94

214214_s_at	C1QBP	708	1.17	9.41	13.38	7.28E-23	4.77E-21	41.54	0.98	9.62	1.86	0.93	0.94	0.91
214598_at	CLDN8	9073	-3.70	9.77	-13.00	3.77E-22	2.11E-20	39.90	0.97	10.50	1.86	0.93	0.91	0.94
216219_at	AQP6	363	3.49	7.95	18.03	3.33E-31	1.47E-28	60.60	0.99	8.29	1.94	0.98	0.94	1.00
216834_at	RGS1	5996	-1.51	6.33	-10.23	1.29E-16	2.69E-15	27.23	0.94	6.15	1.73	0.87	0.87	0.86
217127_at	CTH	1491	1.48	6.23	11.11	2.09E-18	5.99E-17	31.32	0.93	6.07	1.82	0.92	0.86	0.96
217989_at	HSD17B11	51170	1.77	8.38	9.78	1.07E-15	1.91E-14	25.12	0.93	8.22	1.76	0.87	0.97	0.79
218025_s_at	ECI2	10455	1.15	10.29	10.89	5.78E-18	1.52E-16	30.31	0.96	10.72	1.84	0.93	0.86	0.98
218113_at	CEMIP2	23670	-1.24	8.96	-11.26	1.04E-18	3.11E-17	32.02	0.95	8.68	1.89	0.95	1.00	0.89
218186_at	RAB25	57111	-1.97	8.53	-10.79	9.52E-18	2.41E-16	29.82	0.91	8.16	1.78	0.90	1.00	0.78
218191_s_at	LMBRD1	55788	1.08	10.87	9.97	4.44E-16	8.50E-15	26.00	0.95	10.87	1.80	0.89	0.94	0.85
218311_at	MAP4K3	8491	1.66	8.71	19.79	4.61E-34	4.88E-31	67.12	1.00	8.91	2.00	1.00	1.00	1.00
218404_at	SNX10	29887	1.30	8.85	10.55	2.90E-17	6.76E-16	28.71	0.96	9.08	1.81	0.90	0.92	0.89
218559_s_at	MAFB	9935	-1.06	9.03	-9.31	1.00E-14	1.53E-13	22.90	0.92	8.75	1.72	0.87	0.91	0.81
218717_s_at	P3H2	55214	-1.50	9.55	-13.41	6.34E-23	4.19E-21	41.67	0.98	9.49	1.91	0.95	0.91	1.00
218723_s_at	RGCC	28984	1.59	9.68	9.40	6.53E-15	1.02E-13	23.33	0.94	9.90	1.80	0.89	0.94	0.85
218780_at	HOOK2	29911	-1.34	9.46	-25.08	1.02E-41	5.42E-38	84.46	1.00	9.42	2.00	1.00	1.00	1.00
218792_s_at	BSPRY	54836	-1.25	7.83	-17.63	1.52E-30	6.02E-28	59.10	1.00	7.82	2.00	1.00	1.00	1.00
218910_at	ANO10	55129	-1.42	9.71	-14.54	4.64E-25	4.79E-23	46.56	0.99	9.71	1.91	0.95	0.94	0.97
218921_at	SIGIRR	59307	-1.89	8.88	-14.72	2.17E-25	2.36E-23	47.32	0.99	8.87	1.93	0.96	0.96	0.97
218946_at	NFU1	27247	1.09	8.58	12.61	2.24E-21	1.10E-19	38.13	0.98	8.67	1.90	0.95	0.94	0.96
218980_at	FHOD3	80206	-1.02	9.81	-9.89	6.29E-16	1.17E-14	25.65	0.93	9.69	1.83	0.92	0.94	0.89
219179_at	DACT1	51339	-1.51	7.06	-11.04	2.87E-18	8.00E-17	31.01	0.93	7.06	1.80	0.90	0.91	0.89
219196_at	SCG3	29106	-1.48	5.39	-6.48	5.20E-09	3.22E-08	9.88	0.96	5.03	1.85	0.92	0.85	1.00
219305_x_at	FBXO2	26232	-1.06	7.69	-12.64	1.95E-21	9.62E-20	38.27	0.97	7.54	1.92	0.96	0.98	0.94
219382_at	SERTAD3	29946	-1.05	8.87	-17.94	4.59E-31	1.97E-28	60.29	0.99	8.73	1.94	0.98	1.00	0.94
219388_at	GRHL2	79977	-1.02	6.77	-9.81	9.35E-16	1.69E-14	25.26	0.94	6.84	1.82	0.90	0.85	0.97
219411_at	ELMO3	79767	-1.13	7.97	-18.83	1.58E-32	1.01E-29	63.62	1.00	7.86	2.00	1.00	1.00	1.00
219440_at	RAI2	10742	-1.05	9.02	-14.88	1.10E-25	1.27E-23	47.99	0.98	8.96	1.89	0.94	0.91	0.97
219474_at	C3orf52	79669	-1.11	7.16	-10.11	2.30E-16	4.64E-15	26.65	0.92	6.76	1.71	0.87	0.94	0.78
219580_s_at	TMC5	79838	-1.72	5.58	-17.23	7.25E-30	2.26E-27	57.55	1.00	5.21	2.00	1.00	1.00	1.00
219732_at	PLPPR1	54886	2.35	7.64	16.28	3.28E-28	6.92E-26	53.77	1.00	8.05	2.00	1.00	1.00	1.00
219786_at	TESMIN	9633	-1.22	7.53	-12.24	1.19E-20	4.84E-19	36.47	0.97	7.44	1.85	0.93	0.94	0.92
219949_at	LRRC2	79442	1.47	5.59	11.40	5.52E-19	1.73E-17	32.65	0.94	5.74	1.80	0.90	0.89	0.91
221547_at	PRPF18	8559	1.01	7.47	12.38	6.12E-21	2.67E-19	37.13	0.98	7.57	1.85	0.93	0.89	0.96
221823_at	MACIR	90355	-1.62	8.43	-14.75	1.91E-25	2.10E-23	47.44	0.98	8.33	1.92	0.96	0.98	0.94
221880_s_at	FAM174B	400451	-1.09	7.00	-8.46	5.61E-13	6.47E-12	18.91	0.95	6.58	1.86	0.94	1.00	0.86
222014_x_at	MTO1	25821	1.06	7.93	13.64	2.30E-23	1.65E-21	42.68	0.99	7.91	1.94	0.96	1.00	0.94
222243_s_at	TOB2	10766	-1.00	8.59	-10.08	2.56E-16	5.11E-15	26.54	0.96	8.52	1.79	0.89	0.87	0.92
222574_s_at	DHX40	79665	1.06	8.50	10.43	4.95E-17	1.12E-15	28.18	0.94	8.55	1.82	0.90	0.97	0.85
222805_at	MANEA	79694	1.12	5.55	19.00	8.45E-33	6.39E-30	64.24	1.00	5.70	1.98	0.99	1.00	0.98
222984_at	PAIP2	51247	1.04	9.86	9.21	1.57E-14	2.31E-13	22.46	0.91	9.84	1.73	0.87	0.86	0.87
223087_at	ECHDC1	55862	1.08	8.88	10.17	1.70E-16	3.48E-15	26.95	0.95	8.91	1.76	0.88	0.89	0.87
223170_at	TMEM98	26022	1.15	9.22	10.93	4.83E-18	1.29E-16	30.49	0.94	9.32	1.87	0.94	0.92	0.96
223343_at	MS4A7	58475	-1.50	7.81	-13.77	1.30E-23	9.84E-22	43.25	1.00	7.51	1.98	0.99	0.98	1.00
223748_at	SLC4A11	83959	-1.10	7.13	-13.16	1.90E-22	1.12E-20	40.58	0.96	6.90	1.92	0.96	0.98	0.94
224650_at	MAL2	114569	-3.48	9.50	-12.35	7.25E-21	3.07E-19	36.96	0.95	9.76	1.79	0.90	0.96	0.83
225065_x_at	SNHG29	125144	1.26	10.86	16.43	1.77E-28	4.06E-26	54.38	0.99	10.85	1.93	0.96	0.97	0.96
225291_at	PNPT1	87178	1.01	8.20	21.85	3.38E-37	8.95E-34	74.24	1.00	8.38	2.00	1.00	1.00	1.00

225353_s_at	C1QC	714	-1.03	8.88	-8.79	1.14E-13	1.48E-12	20.49	0.93	8.62	1.79	0.90	0.96	0.83
225835_at	SLC12A2	6558	-1.09	7.99	-10.94	4.74E-18	1.27E-16	30.51	0.97	7.92	1.91	0.95	0.94	0.97
225846_at	ESRP1	54845	-2.28	7.75	-19.80	4.56E-34	4.88E-31	67.13	1.00	7.99	1.98	0.99	0.98	1.00
225897_at	MARCKS	4082	1.47	8.28	11.24	1.17E-18	3.51E-17	31.90	0.94	8.51	1.81	0.92	0.83	0.98
225911_at	NPNT	255743	-2.09	8.28	-15.48	8.91E-27	1.30E-24	50.49	0.99	8.46	1.91	0.95	0.94	0.97
226068_at	SYK	6850	-1.47	7.28	-13.38	7.24E-23	4.77E-21	41.54	0.98	7.13	1.87	0.94	0.98	0.89
226129_at	FAM83H	286077	-1.36	8.19	-16.44	1.72E-28	4.02E-26	54.41	1.00	8.04	1.98	0.99	0.98	1.00
226185_at	CDS1	1040	-1.61	8.68	-12.72	1.34E-21	6.90E-20	38.64	0.97	8.86	1.84	0.92	0.89	0.94
226403_at	TMC4	147798	-1.59	9.84	-15.07	4.76E-26	6.00E-24	48.83	0.99	9.94	1.91	0.95	0.94	0.97
226748_at	LYSMD2	256586	-1.35	9.19	-12.50	3.67E-21	1.69E-19	37.64	0.96	9.07	1.94	0.98	1.00	0.94
226824_at	CPXM2	119587	-1.74	8.99	-8.73	1.51E-13	1.92E-12	20.21	0.90	8.10	1.73	0.88	0.98	0.75
226913_s_at	SOX8	30812	1.06	5.47	10.60	2.28E-17	5.41E-16	28.95	0.94	5.49	1.83	0.90	1.00	0.83
226939_at	CPEB2	132864	-1.01	9.27	-13.24	1.31E-22	8.08E-21	40.95	0.97	9.16	1.81	0.92	0.98	0.83
227015_at	ASPHD2	57168	-1.61	7.38	-12.82	8.42E-22	4.49E-20	39.10	0.96	7.49	1.80	0.89	0.85	0.94
227048_at	LAMA1	284217	-1.90	6.46	-13.99	4.94E-24	4.06E-22	44.21	0.97	5.87	1.87	0.94	0.96	0.92
227291_s_at	BOLA3	388962	1.23	9.89	13.02	3.56E-22	2.01E-20	39.96	0.97	10.16	1.92	0.96	0.94	0.98
227471_at	HACE1	57531	1.04	6.78	10.79	9.44E-18	2.40E-16	29.83	0.95	6.81	1.80	0.90	0.86	0.94
228186_s_at	RSPO3	84870	-1.74	7.32	-13.19	1.64E-22	9.84E-21	40.73	0.99	6.89	1.96	0.98	0.96	1.00
228338_at	COLCA2	120376	-1.42	7.95	-10.80	8.90E-18	2.28E-16	29.89	0.95	7.70	1.89	0.95	1.00	0.89
228697_at	HINT3	135114	1.00	5.66	9.93	5.36E-16	1.01E-14	25.81	0.96	5.48	1.79	0.88	1.00	0.79
229057_at	SCN2A	6326	1.22	6.60	9.43	5.63E-15	8.88E-14	23.47	0.93	6.71	1.83	0.92	0.89	0.94
229070_at	ADTRP	84830	-1.25	6.91	-12.54	3.08E-21	1.45E-19	37.81	0.97	6.70	1.87	0.94	0.98	0.89
229309_at	ADRB1	153	1.89	5.58	14.50	5.35E-25	5.45E-23	46.42	1.00	5.95	1.97	0.99	0.97	1.00
229396_at	OVOL1	5017	-1.10	7.77	-16.23	4.07E-28	8.39E-26	53.55	1.00	7.78	1.93	0.96	0.96	0.97
229649_at	NRXN3	9369	-1.87	7.94	-9.14	2.26E-14	3.23E-13	22.09	0.92	7.56	1.78	0.90	0.98	0.81
229797_at	MCOLN3	55283	2.44	7.97	15.90	1.54E-27	2.81E-25	52.23	0.99	8.13	1.98	0.99	1.00	0.98
229831_at	CNTN3	5067	-2.44	7.55	-14.12	2.79E-24	2.47E-22	44.78	0.98	7.91	1.87	0.93	0.89	0.97
229860_x_at	C4orf48	401115	-1.28	7.92	-8.98	4.64E-14	6.36E-13	21.38	0.91	7.75	1.85	0.93	0.94	0.92
230110_at	MCOLN2	255231	2.49	6.84	15.75	2.87E-27	4.80E-25	51.61	0.97	6.97	1.90	0.95	0.92	0.98
230174_at	LYPLAL1	127018	1.04	9.78	14.22	1.86E-24	1.73E-22	45.18	0.99	9.99	1.90	0.95	0.92	0.98
230237_at	ADCYAP1	116	-1.13	5.88	-10.97	4.03E-18	1.10E-16	30.67	0.98	5.53	1.90	0.95	0.98	0.92
230560_at	STXBP6	29091	-2.82	7.18	-13.62	2.50E-23	1.77E-21	42.60	0.98	6.41	1.89	0.95	1.00	0.89
230644_at	LRFN5	145581	-2.02	6.36	-13.24	1.35E-22	8.27E-21	40.92	0.97	6.31	1.94	0.96	0.94	1.00
230883_at	NXPH2	11249	1.79	6.18	12.07	2.56E-20	1.00E-18	35.70	0.96	6.03	1.80	0.89	0.97	0.83
231867_at	TENM2	57451	2.03	6.20	15.86	1.79E-27	3.23E-25	52.08	0.99	6.24	1.96	0.98	1.00	0.96
233183_at	SLC4A9	83697	1.75	7.97	13.46	4.90E-23	3.28E-21	41.93	0.98	8.22	1.86	0.94	0.86	1.00
233642_s_at	HEATR5B	54497	1.03	7.88	15.96	1.20E-27	2.27E-25	52.48	1.00	7.92	1.96	0.98	1.00	0.96
234970_at	TC2N	123036	-1.20	5.61	-12.20	1.41E-20	5.67E-19	36.30	0.96	5.74	1.82	0.90	0.87	0.94
235046_at	INPP4B	8821	-2.20	7.21	-10.16	1.84E-16	3.75E-15	26.87	0.93	6.86	1.74	0.88	0.96	0.78
235198_at	OSTM1	28962	1.03	6.29	14.85	1.25E-25	1.41E-23	47.87	0.98	6.29	1.91	0.95	0.97	0.94
236356_at	NDUFS1	4719	1.15	9.05	17.32	5.24E-30	1.76E-27	57.87	0.99	9.18	1.97	0.99	0.97	1.00
236489_at	ADGRF1	266977	1.43	6.58	11.50	3.55E-19	1.14E-17	33.09	0.95	6.50	1.76	0.87	0.97	0.79
238513_at	PRRG4	79056	-1.16	7.50	-12.55	2.90E-21	1.37E-19	37.87	0.98	7.51	1.84	0.92	0.87	0.97
238763_at	RBM20	282996	1.39	5.45	10.00	3.81E-16	7.36E-15	26.15	0.95	5.23	1.79	0.89	0.92	0.87
238815_at	LRRTM1	347730	-1.28	7.02	-9.41	6.30E-15	9.83E-14	23.36	0.95	6.68	1.83	0.92	0.94	0.89
238865_at	PABPC4L	132430	-1.85	7.28	-16.28	3.27E-28	6.92E-26	53.77	0.99	7.36	1.98	0.99	0.98	1.00
239183_at	ANGPTL1	9068	1.06	6.22	8.35	9.07E-13	1.01E-11	18.43	0.90	6.10	1.66	0.82	0.92	0.74
240117_at	FBN3	84467	-1.14	7.22	-8.51	4.35E-13	5.12E-12	19.16	0.91	7.25	1.68	0.83	0.79	0.89

240395_at	DGKI	9162	-1.39	6.44	-7.59	3.21E-11	2.80E-10	14.90	0.91	6.17	1.75	0.87	0.81	0.94
241859_at	PLCL1	5334	1.77	8.33	11.98	3.91E-20	1.48E-18	35.28	0.95	8.52	1.92	0.96	0.94	0.98
243087_at	DNAI3	126820	1.16	5.25	12.44	4.78E-21	2.14E-19	37.37	0.97	5.23	1.88	0.94	0.94	0.94
65517_at	AP1M2	10053	-1.49	8.84	-17.31	5.33E-30	1.76E-27	57.85	1.00	8.87	1.96	0.98	0.96	1.00

Table S3: Reactome Pathway analysis between chRCC and RO using all genes (n = 15,875)

Reactome pathways	Description	GeneRatio	p.adjust	qvalue	Count
R-HSA-71387	Metabolism of carbohydrates	282/9216	5.93E-07	3.58E-07	282
R-HSA-3700989	Transcriptional Regulation by TP53	341/9216	3.18E-05	1.92E-05	341
R-HSA-76002	Platelet activation, signaling and aggregation	248/9216	7.74E-05	4.67E-05	248
R-HSA-5663202	Diseases of signal transduction by growth factor receptors and second messengers	366/9216	7.74E-05	4.67E-05	366
R-HSA-112316	Neuronal System	378/9216	1.81E-04	1.09E-04	378
R-HSA-397014	Muscle contraction	187/9216	2.63E-04	1.59E-04	187
R-HSA-1630316	Glycosaminoglycan metabolism	120/9216	4.98E-04	3.00E-04	120
R-HSA-1474244	Extracellular matrix organization	279/9216	6.40E-04	3.86E-04	279
R-HSA-6785807	Interleukin-4 and Interleukin-13 signaling	105/9216	6.40E-04	3.86E-04	105
R-HSA-3858494	Beta-catenin independent WNT signaling	139/9216	1.55E-03	9.33E-04	139
R-HSA-72172	mRNA Splicing	179/9216	2.29E-03	1.38E-03	179
R-HSA-5357801	Programmed Cell Death	196/9216	2.29E-03	1.38E-03	196
R-HSA-400206	Regulation of lipid metabolism by PPAR-alpha	114/9216	2.29E-03	1.38E-03	114
R-HSA-5576891	Cardiac conduction	123/9216	2.36E-03	1.43E-03	123
R-HSA-1989781	PPARA activates gene expression	112/9216	2.45E-03	1.48E-03	112
R-HSA-6798695	Neutrophil degranulation	434/9216	2.45E-03	1.48E-03	434
R-HSA-72163	mRNA Splicing - Major Pathway	171/9216	3.69E-03	2.23E-03	171
R-HSA-1852241	Organelle biogenesis and maintenance	271/9216	4.24E-03	2.56E-03	271
R-HSA-112315	Transmission across Chemical Synapses	248/9216	4.24E-03	2.56E-03	248
R-HSA-109581	Apoptosis	168/9216	4.24E-03	2.56E-03	168
R-HSA-8951664	Neddylation	216/9216	4.28E-03	2.58E-03	216
R-HSA-5633007	Regulation of TP53 Activity	150/9216	4.28E-03	2.58E-03	150
R-HSA-9006925	Intracellular signaling by second messengers	282/9216	4.90E-03	2.96E-03	282
R-HSA-449147	Signaling by Interleukins	416/9216	5.48E-03	3.30E-03	416
R-HSA-72203	Processing of Capped Intron-Containing Pre-mRNA	224/9216	6.92E-03	4.17E-03	224
R-HSA-196854	Metabolism of vitamins and cofactors	176/9216	7.06E-03	4.26E-03	176
R-HSA-76005	Response to elevated platelet cytosolic Ca2+	126/9216	7.06E-03	4.26E-03	126
R-HSA-425407	SLC-mediated transmembrane transport	230/9216	7.06E-03	4.26E-03	230
R-HSA-9006936	Signaling by TGFB family members	97/9216	8.14E-03	4.91E-03	97
R-HSA-73887	Death Receptor Signaling	132/9216	8.37E-03	5.05E-03	132
R-HSA-446203	Asparagine N-linked glycosylation	276/9216	9.97E-03	6.02E-03	276
R-HSA-114608	Platelet degranulation	121/9216	0.01	6.12E-03	121
R-HSA-163685	Integration of energy metabolism	102/9216	0.011	6.93E-03	102
R-HSA-8856828	Clathrin-mediated endocytosis	136/9216	0.011	6.93E-03	136
R-HSA-1257604	PIP3 activates AKT signaling	243/9216	0.012	7.12E-03	243
R-HSA-416476	G alpha (q) signaling events	198/9216	0.012	7.12E-03	198
R-HSA-166520	Signaling by NTRKs	125/9216	0.013	0.0081	125
R-HSA-2454202	Fc epsilon receptor (FCERI) signaling	125/9216	0.013	0.0081	125
R-HSA-9658195	Leishmania infection	231/9216	0.015	0.0091	231
R-HSA-983168	Antigen processing: Ubiquitination & Proteasome degradation	279/9216	0.016	0.0095	279
R-HSA-9012999	RHO GTPase cycle	396/9216	0.016	0.0095	396
R-HSA-5683057	MAPK family signaling cascades	293/9216	0.016	0.0095	293
R-HSA-453279	Mitotic G1 phase and G1/S transition	138/9216	0.016	0.0095	138

R-HSA-5358351	Signaling by Hedgehog	138/9216	0.016	0.0095	138
R-HSA-983705	Signaling by the B Cell Receptor (BCR)	105/9216	0.016	0.0095	105
R-HSA-162906	HIV Infection	213/9216	0.017	0.01	213
R-HSA-1500931	Cell-Cell communication	120/9216	0.017	0.011	120
R-HSA-9679506	SARS-CoV Infections	136/9216	0.017	0.011	136
R-HSA-983169	Class I MHC mediated antigen processing & presentation	338/9216	0.017	0.011	338
R-HSA-9013149	RAC1 GTPase cycle	169/9216	0.026	0.016	169
R-HSA-948021	Transport to the Golgi and subsequent modification	169/9216	0.026	0.016	169
R-HSA-187037	Signaling by NTRK1 (TRKA)	107/9216	0.026	0.016	107
R-HSA-5617833	Cilium Assembly	183/9216	0.027	0.016	183
R-HSA-196849	Metabolism of water-soluble vitamins and cofactors	114/9216	0.028	0.017	114
R-HSA-1474228	Degradation of the extracellular matrix	129/9216	0.028	0.017	129
R-HSA-6807070	PTEN Regulation	129/9216	0.028	0.017	129
R-HSA-418594	G alpha (i) signaling events	285/9216	0.031	0.018	285
R-HSA-1296071	Potassium Channels	96/9216	0.031	0.018	96
R-HSA-8878166	Transcriptional regulation by RUNX2	112/9216	0.031	0.018	112
R-HSA-500792	GPCR ligand binding	414/9216	0.037	0.022	414
R-HSA-983712	Ion channel transport	169/9216	0.039	0.023	169
R-HSA-8957322	Metabolism of steroids	138/9216	0.043	0.026	138
R-HSA-381426	Regulation of Insulin-like Growth Factor (IGF) transport and uptake by Insulin-like Growth Factor Binding Proteins (IGFBPs)	115/9216	0.044	0.027	115
R-HSA-5619115	Disorders of transmembrane transporters	159/9216	0.045	0.027	159
R-HSA-72306	tRNA processing	99/9216	0.045	0.027	99
R-HSA-5684996	MAPK1/MAPK3 signaling	256/9216	0.046	0.028	256
R-HSA-8856825	Cargo recognition for clathrin-mediated endocytosis	98/9216	0.048	0.029	98

Table S5: KEGG pathway analysis of genes (n = 15,875) between RO and Normal Kidney

ID	Description	setSize	enrichmentSc	NES	pvalue	p.adjust	qvalues	rank
hsa00190	Oxidative phosphorylation	112	0.71450272	2.79627759	1.73E-19	3.74E-17	2.90E-17	2850
hsa04714	Thermogenesis	204	0.52918591	2.2126015	1.26E-12	1.36E-10	1.05E-10	2672
hsa04932	Non-alcoholic fatty liver disease	143	0.50531716	2.01419525	4.99E-08	3.59E-06	2.78E-06	2626
hsa04610	Complement and coagulation cascades	73	-0.6696888	-1.9819239	1.92E-06	8.74E-05	6.77E-05	1590
hsa05418	Fluid shear stress and atherosclerosis	125	-0.5910539	-1.8912175	2.02E-06	8.74E-05	6.77E-05	3216
hsa05415	Diabetic cardiomyopathy	177	0.42852604	1.76720808	3.40E-06	1.22E-04	9.47E-05	2850
hsa04020	Calcium signaling pathway	226	0.39633067	1.69038892	6.52E-06	2.01E-04	1.56E-04	1040
hsa05010	Alzheimer disease	330	0.34873166	1.53068077	1.23E-05	3.21E-04	2.49E-04	2850
hsa04978	Mineral absorption	53	-0.7001771	-1.9929178	1.37E-05	3.21E-04	2.49E-04	1936
hsa04974	Protein digestion and absorption	90	-0.605544	-1.8521446	1.57E-05	3.21E-04	2.49E-04	1936
hsa04514	Cell adhesion molecules	124	-0.565881	-1.8120234	1.63E-05	3.21E-04	2.49E-04	2492
hsa05150	Staphylococcus aureus infection	66	-0.6381053	-1.8666056	3.40E-05	6.12E-04	4.74E-04	1571
hsa05016	Huntington disease	265	0.35487518	1.52776849	4.32E-05	7.17E-04	5.56E-04	3277
hsa04670	Leukocyte transendothelial migration	99	-0.586234	-1.8161851	4.82E-05	7.44E-04	5.76E-04	3216
hsa05014	Amyotrophic lateral sclerosis	313	0.33578125	1.47830791	5.64E-05	8.13E-04	6.30E-04	2672
hsa05012	Parkinson disease	216	0.37728942	1.60530172	6.81E-05	9.20E-04	7.13E-04	2342
hsa05322	Systemic lupus erythematosus	81	-0.5991339	-1.8074699	7.65E-05	9.73E-04	7.54E-04	1088
hsa00980	Metabolism of xenobiotics by cytochrome P450	51	-0.6661358	-1.879733	8.41E-05	0.00100946	7.82E-04	1013
hsa04668	TNF signaling pathway	106	-0.5701657	-1.7890246	9.01E-05	0.00102416	7.94E-04	2719
hsa05133	Pertussis	68	-0.6210587	-1.8268115	1.19E-04	0.00128167	9.93E-04	2405
hsa05130	Pathogenic Escherichia coli infection	173	-0.5136957	-1.6978514	1.32E-04	0.0013569	0.0010514	3074
hsa04512	ECM-receptor interaction	82	-0.58691	-1.7713702	1.76E-04	0.00173003	0.00134052	1956
hsa05022	Pathways of neurodegeneration - multiple diseases	423	0.28996118	1.29488092	4.93E-04	0.00451916	0.00350169	2850
hsa05020	Prion disease	239	0.3423339	1.47722924	5.02E-04	0.00451916	0.00350169	2851
hsa04723	Retrograde endocannabinoid signaling	133	0.39492796	1.5839345	5.41E-04	0.00459413	0.00355978	2586
hsa00983	Drug metabolism - other enzymes	55	-0.6149623	-1.7588473	5.53E-04	0.00459413	0.00355978	1685
hsa04721	Synaptic vesicle cycle	73	0.47141536	1.73755962	9.81E-04	0.0078458	0.00607935	785
hsa05231	Choline metabolism in cancer	91	-0.5378764	-1.6471865	0.00122009	0.00941211	0.00729301	1945
hsa04976	Bile secretion	74	-0.5440012	-1.6189053	0.00157244	0.01171195	0.00907505	662
hsa00010	Glycolysis / Gluconeogenesis	64	-0.579041	-1.6928824	0.00164232	0.01182473	0.00916243	2038
hsa05145	Toxoplasmosis	100	-0.5116571	-1.58819	0.00207129	0.01443222	0.01118286	3131
hsa04740	Olfactory transduction	108	0.39372511	1.5280235	0.00228074	0.015395	0.01192888	3473
hsa01230	Biosynthesis of amino acids	66	-0.5522244	-1.6153843	0.00261843	0.01713881	0.01328007	1237
hsa04146	Peroxisome	76	-0.524959	-1.571039	0.00340743	0.0216472	0.01677342	2316
hsa05171	Coronavirus disease - COVID-19	170	-0.4640824	-1.5308589	0.00376237	0.02321917	0.01799147	2946

hsa04530	Tight junction	152	-0.4645904	-1.5206929	0.00459955	0.02759728	0.02138386	2963
hsa05160	Hepatitis C	139	-0.4696928	-1.5173356	0.00546441	0.03190033	0.02471809	3236
hsa04657	IL-17 signaling pathway	87	-0.5238416	-1.5963681	0.00570132	0.03240751	0.02511108	3272
hsa03320	PPAR signaling pathway	69	-0.5267584	-1.5494107	0.00659022	0.03649968	0.02828191	2436
hsa00561	Glycerolipid metabolism	56	-0.5553908	-1.5872777	0.00733531	0.0396107	0.0306925	3010
hsa04510	Focal adhesion	188	-0.4219662	-1.4122021	0.00908993	0.0478884	0.03710651	1873

Table S4: KEGG pathway analysis of genes (n=15875) between chRCC and normal kidney.

ID	Description	setSize	enrichmentSc	NES	pvalue	p.adjust	qvalues	rank
hsa04974	Protein digestion and absorption	90	-0.7	-2.1	1.77E-08	3.82E-06	3.35E-06	2063
hsa04610	Complement and coagulation cascades	73	-0.7	-2.1	8.20E-08	8.86E-06	7.77E-06	874
hsa04146	Peroxisome	76	-0.6	-2.0	4.51E-06	2.70E-04	2.37E-04	1766
hsa00190	Oxidative phosphorylation	112	0.5	2.0	5.00E-06	2.70E-04	2.37E-04	2156
hsa00980	Metabolism of xenobiotics by cytochrome P450	51	-0.7	-2.0	1.04E-05	4.49E-04	3.94E-04	1546
hsa00010	Glycolysis / Gluconeogenesis	64	-0.6	-2.0	1.91E-05	6.88E-04	6.03E-04	1546
hsa03320	PPAR signaling pathway	69	-0.6	-1.9	1.08E-04	0.00332406	0.00291584	1182
hsa01200	Carbon metabolism	109	-0.5	-1.7	1.93E-04	0.00522253	0.00458116	1884
hsa05418	Fluid shear stress and atherosclerosis	125	-0.5	-1.6	4.53E-04	0.01019494	0.00894293	2680
hsa00983	Drug metabolism - other enzymes	55	-0.6	-1.8	4.72E-04	0.01019494	0.00894293	426
hsa05206	MicroRNAs in cancer	155	-0.5	-1.6	6.72E-04	0.01318604	0.0115667	2751
hsa04978	Mineral absorption	53	-0.6	-1.8	0.00118607	0.02134929	0.01872744	2781
hsa01240	Biosynthesis of cofactors	126	-0.5	-1.5	0.00163406	0.02715053	0.02381626	3240
hsa04976	Bile secretion	74	-0.5	-1.7	0.00225048	0.03386365	0.02970496	1824
hsa04932	Non-alcoholic fatty liver disease	143	0.4	1.5	0.00235164	0.03386365	0.02970496	2156
hsa04512	ECM-receptor interaction	82	-0.5	-1.7	0.00282432	0.0381283	0.03344588	2074
hsa05323	Rheumatoid arthritis	77	0.4	1.6	0.0034201	0.04119485	0.03613583	1677
hsa01230	Biosynthesis of amino acids	66	-0.5	-1.7	0.0034329	0.04119485	0.03613583	1377
hsa04070	Phosphatidylinositol signaling system	96	0.4	1.6	0.00391509	0.04450842	0.03904247	2127

Material

1.1 Figure Ss

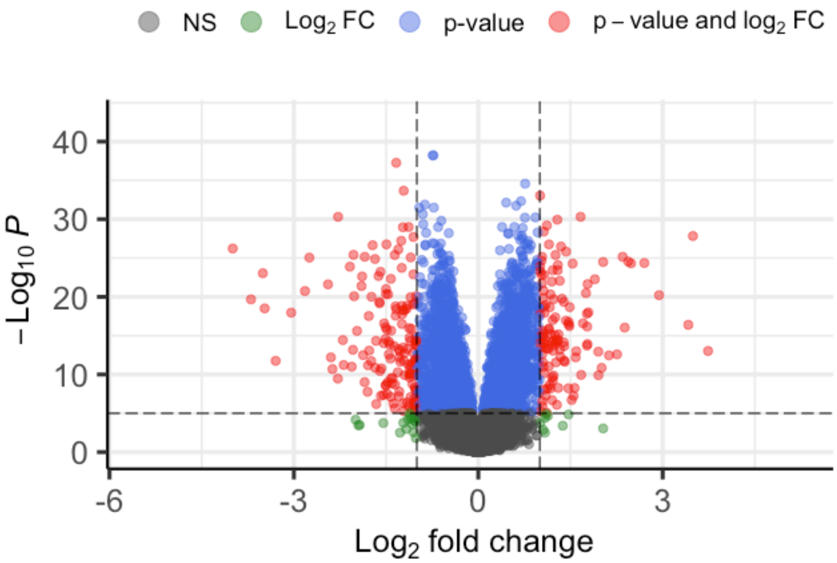

Figure S1: Differential expression analysis between chRCC and RO tumor types using 15875 genes. Differential expression was performed on log2 transformed data using LIMMA package in R. cutoffs for p-value (horizontal line) is $1e^{-5}$ and log2 fold change (vertical lines) is 1. Genes marked with red color represent candidate markers for gene signature based on differential expression.

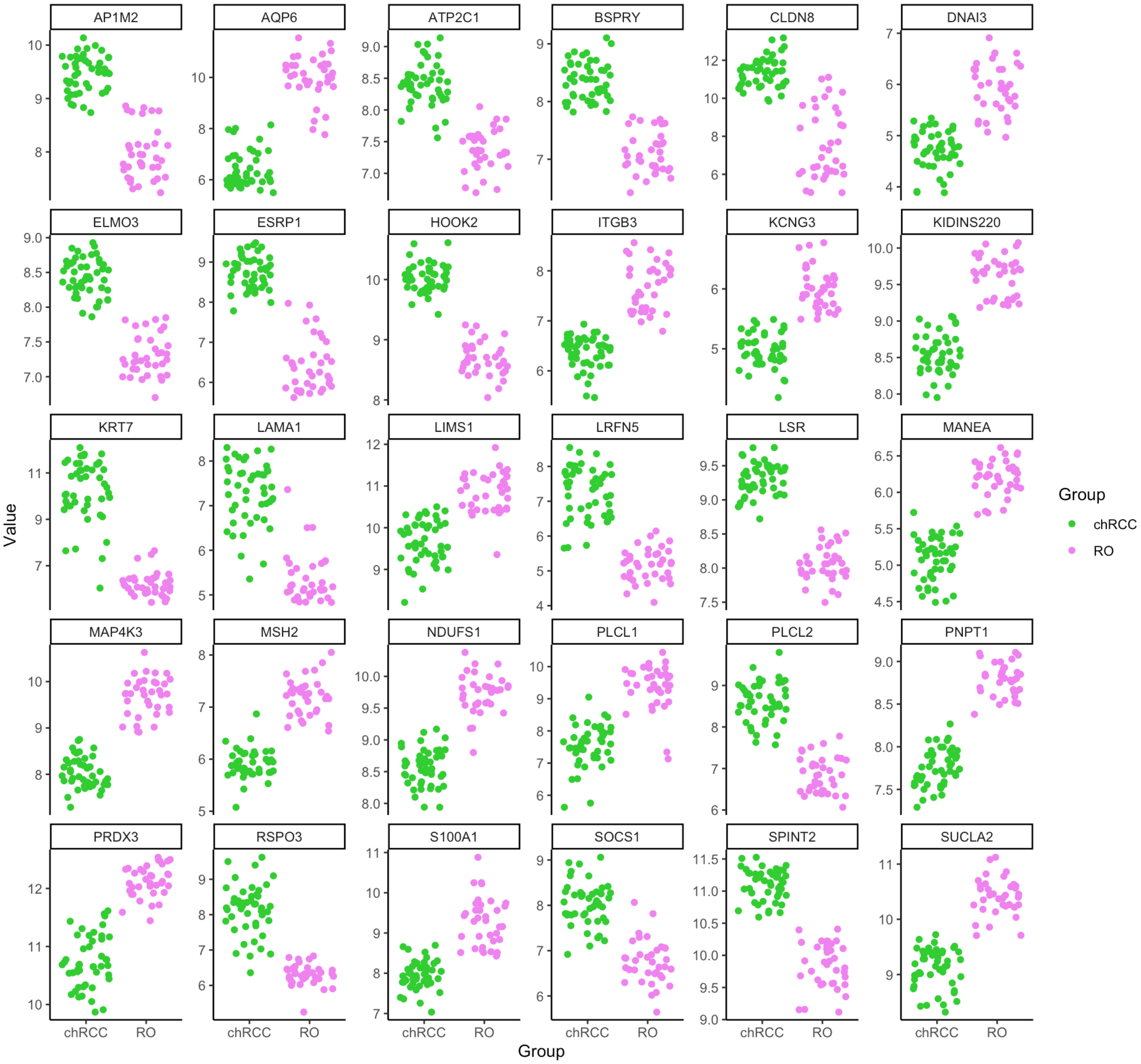

Figure S2: Dot plots for each gene in COGS for classification in chRCC (green dots) and renal oncocytoma (magenta dots). X-axes represent histology and y-axes represents Log2 expression.

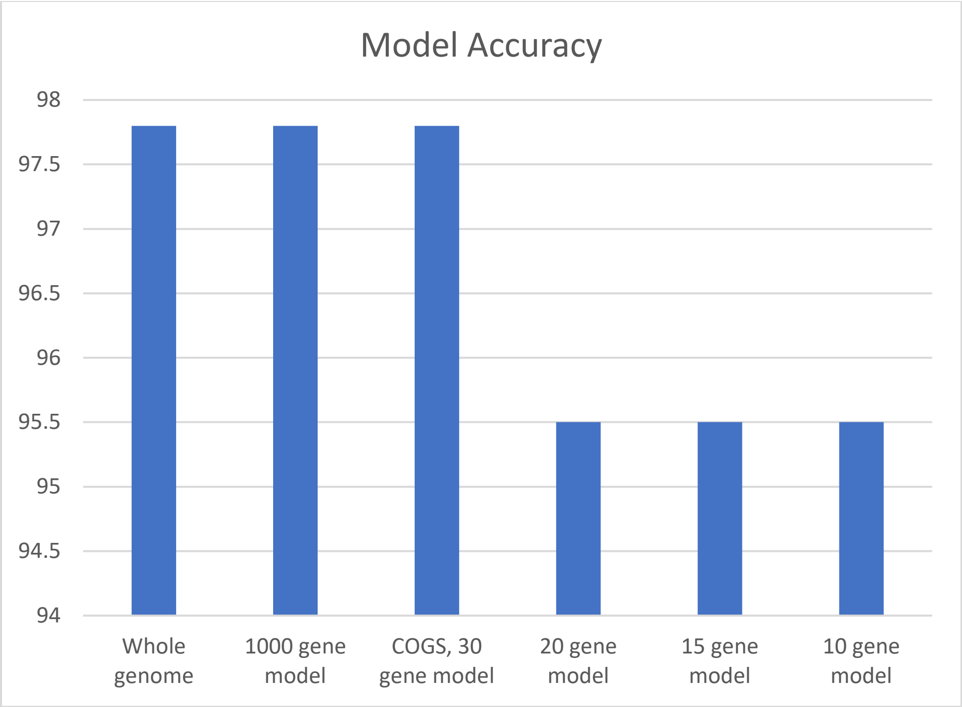

Figure S3: Model performance from whole genome to 10 gene model. Accuracy was checked with density based UMAP classification.

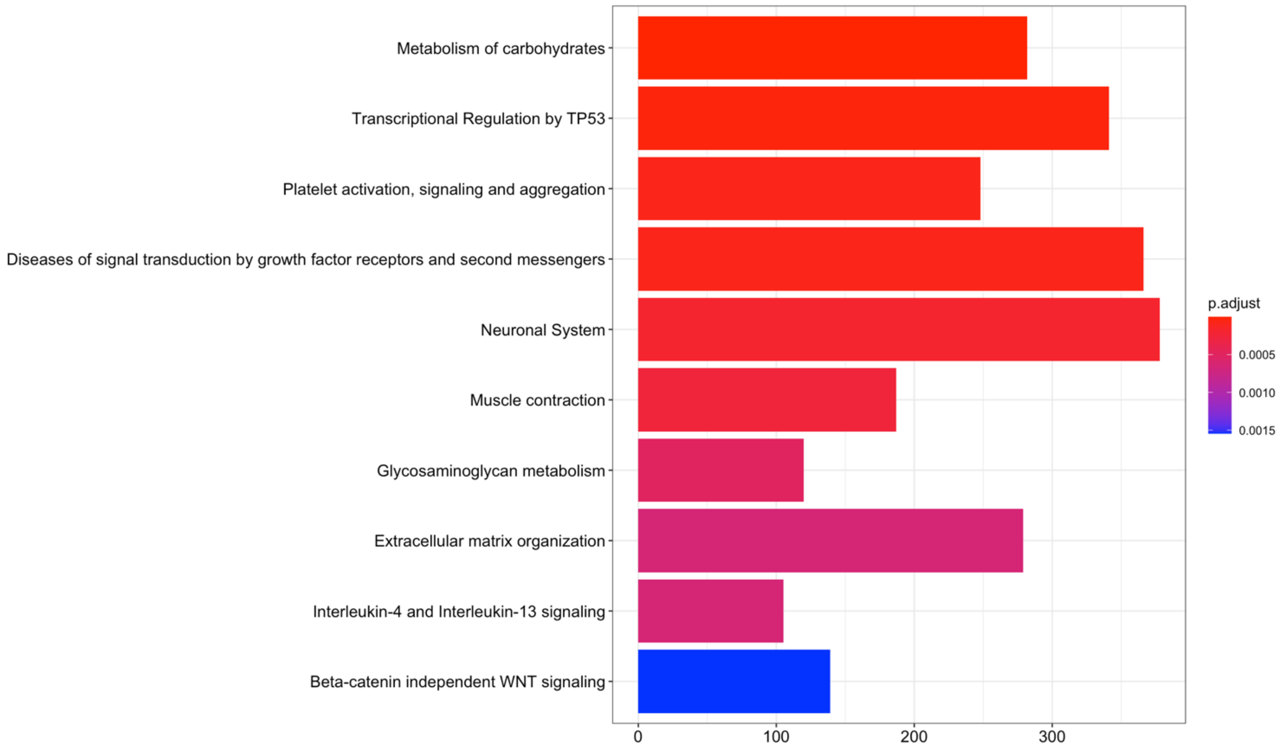

Figure S4: Reactome pathways analysis between chRCC and RO for all genes (15,875 genes) showing enrichment of specific pathways. X-axes represents number of genes present in each pathway.

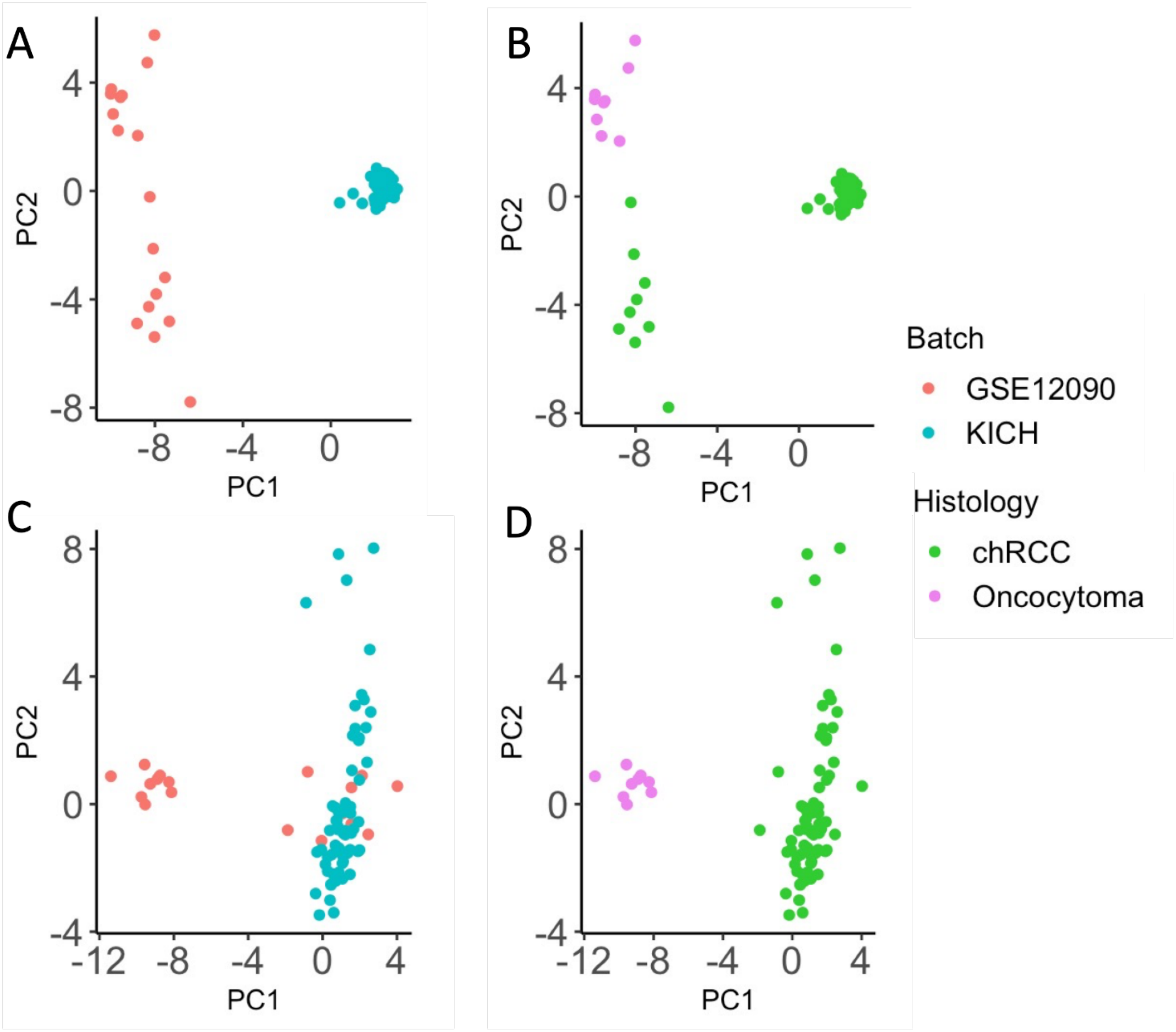

Figure S5: Principal component analysis showing batch effects (A-B) and after correction (C-D) on the validation dataset.
